# Altered distribution and function of NK-cell subsets lead to impaired tumor surveillance in JAK2V617F myeloproliferative neoplasms

**DOI:** 10.3389/fimmu.2022.768592

**Published:** 2022-09-23

**Authors:** Amanda Fernandes de Oliveira Costa, Leticia Olops Marani, Thiago Mantello Bianco, Adriana Queiroz Arantes, Izabela Aparecida Lopes, Diego Antonio Pereira-Martins, Leonardo Carvalho Palma, Priscila Santos Scheucher, Josiane Lilian dos Santos Schiavinato, Larissa Sarri Binelli, Cleide Araújo Silva, Susumu S. Kobayashi, João Agostinho Machado-Neto, Eduardo Magalhães Rego, Robert Samuel Welner, Lorena Lobo de Figueiredo-Pontes

**Affiliations:** ^1^ Division of Hematology, Department of Medical Imaging, Hematology, and Oncology, Ribeirão Preto Medical School, University of São Paulo, Ribeirão Preto, Brazil; ^2^ Center for Cell-based Therapy, Regional Blood Center of Ribeirão Preto, Ribeirão Preto Medical School, University of São Paulo, Ribeirao Preto, Brazil; ^3^ Department of Medicine, Beth Israel Deaconess Medical Center, Harvard Medical School, Boston, MA, United States; ^4^ Division of Translational Genomics, Exploratory Oncology Research, and Clinical Trial Center, National Cancer Center, Kashiwa, Japan; ^5^ Department of Pharmacology, Institute of Biomedical Sciences, University of São Paulo, São Paulo, Brazil; ^6^ Division of Hematology, University of São Paulo Medical School, São Paulo, Brazil; ^7^ Division Hematology/Oncology, School of Medicine, University of Alabama at Birmingham, Birmingham, AL, United States

**Keywords:** myeloproliferative neoplasms, JAK2v617F mutation, natural killer cells, natural killer cells subsets, flow cytometry

## Abstract

In cancer, tumor cells and their neoplastic microenvironment can sculpt the immunogenic phenotype of a developing tumor. In this context, natural killer (NK) cells are subtypes of lymphocytes of the innate immune system recognized for their potential to eliminate neoplastic cells, not only through direct cytolytic activity but also by favoring the development of an adaptive antitumor immune response. Even though the protective effect against leukemia due to NK-cell alloreactivity mediated by the absence of the KIR-ligand has already been shown, and some data on the role of NK cells in myeloproliferative neoplasms (MPN) has been explored, their mechanisms of immune escape have not been fully investigated. It is still unclear whether NK cells can affect the biology of BCR-ABL1-negative MPN and which mechanisms are involved in the control of leukemic stem cell expansion. Aiming to investigate the potential contribution of NK cells to the pathogenesis of MPN, we characterized the frequency, receptor expression, maturation profile, and function of NK cells from a conditional Jak2V617F murine transgenic model, which faithfully resembles the main clinical and laboratory characteristics of human polycythemia vera, and MPN patients. Immunophenotypic analysis was performed to characterize NK frequency, their subtypes, and receptor expression in both mutated and wild-type samples. We observed a higher frequency of total NK cells in JAK2V617F mutated MPN and a maturation arrest that resulted in low-numbered mature CD11b^+^ NK cells and increased immature secretory CD27^+^ cells in both human and murine mutated samples. In agreement, inhibitory receptors were more expressed in MPN. NK cells from Jak2V617F mice presented a lower potential for proliferation and activation than wild-type NK cells. Colonies generated by murine hematopoietic stem cells (HSC) after mutated or wild-type NK co-culture exposure demonstrated that NK cells from Jak2V617F mice were deficient in regulating differentiation and clonogenic capacity. In conclusion, our findings suggest that NK cells have an immature profile with deficient cytotoxicity that may lead to impaired tumor surveillance in MPN. These data provide a new perspective on the behavior of NK cells in the context of myeloid malignancies and can contribute to the development of new therapeutic strategies, targeting onco-inflammatory pathways that can potentially control transformed HSCs.

## 1 Introduction

Natural killer (NK) cells are lymphocytes of the innate immune system with both cytotoxicity and cytokine-producing effector functions (1, 2). Their lytic activity is based on cytotoxic granules release, containing perforin and granzyme, or alternatively, by TNF, FAS ligand, and TRAIL-dependent apoptosis mechanisms that enable them to recognize and kill aberrant cells and rapidly produce soluble factors with antimicrobial effects (3, 4). The regulation of NK effector functions relies on the balance of an arsenal of surface activating and inhibitory receptors, and once activation crosses a given threshold, NK-cell activation proceeds. Their ability to activate receptors by binding their ligands gives them an important role in hematopoiesis, not only eliminating susceptible targets but also recruiting other cells to amplify the inflammatory response (3, 5, 6).

Several coordinated differentiation and maturation steps result in the progressive acquisition of functional competency (7–9). Committed NK cells from mice acquire NK1.1, CD94/NKG2A, and NKp46 followed by CD11b and CD27 (10). Similarly, in humans, they first acquire CD56, CD94/NKG2A, NKp46, and NKG2D, and finally, killer immunoglobulin-like receptors (KIR) and CD16 (11–13) followed by CD11b and CD27. Also, human NK cells can be classified into two subsets with different CD56 cell-surface densities and distinct phenotypic properties (14). CD56^bright^ NK cells are relatively enriched in secondary lymphoid tissues and have high cytokine production capacity but relatively lower cytotoxicity in comparison to CD56^dim^ NK cells that predominate in peripheral blood (PB) and display lower levels of cytokine production but more potent cytolytic capacity against target cells (15, 16).

It is known that tumor cells and the neoplastic microenvironment can modify the NK-cell phenotype reducing its effector function against disease progression (17–20). In bone marrow transplantation (BMT) studies, a protective effect against leukemia has been attributed to NK-cell alloreactivity mediated by the absence of the KIR-ligand (21–24). Evidence of antitumor immunogenicity has been more widely demonstrated in solid tumors, and only more recently, the antitumor effects of NK cells in myeloproliferative neoplasms (MPN) have been investigated. MPN are characterized by exacerbated myeloid proliferation that may result in bone marrow fibrosis and, in some cases, progression to acute leukemia. This group of myeloid neoplasms includes the classic chronic myeloid leukemia (CML), driven by t(9;22)/BCR-ABL1 translocation, and BCR-ABL1-negative MPN, which can be categorized as polycythemia vera (PV), essential thrombocythemia (ET), and primary myelofibrosis (PMF) (22–24). Almost all PV and approximately half of ET and PMF patients harbor a gain-of-function mutation that results in a V617F amino acid change in the JAK2 protein, mediating constitutive phosphorylation and downstream signaling *via* STAT3 and STAT5 (25).

Except for specific tyrosine-kinase inhibitors (TKI) in CML, available therapy for MPN other than BMT has not been proven to be disease-modifying. Therefore, immune system regulation as an alternative approach is certainly very attractive. It is known that NK dysfunction can be restored with TKI in CML, contributing to molecular remission and early recovery of NK cells after BMT (26–29). In BCR-ABL1-negative MPN, however, little is known about their role in disease initiation, progression, or treatment response. An early study suggested that patients with BCR-ABL1-negative MPN frequently have low endogenous NK-cell activity *in vitro*, and Gersuk et al. argued that PMF patients have lower numbers of NK cells when compared to patients with ET and PV. It was also already shown that MPN patients, especially PMF patients, may have reduced NK cytotoxicity that is not reversible by IL-2 addition (30, 31). On the other hand, others have shown an increased proportion of NK cells in PV patients as compared to healthy donors (32), and, more recently, increased CD56^bright^ and decreased CD56^dim^ NK cells were found when MPN patients were treated with IFN-α (33).

All these findings suggest that NK cells play a central role in the control of myeloid malignancies by fighting disease progression. However, available data are often conflicting, and it is still unclear how NK-cell dysfunction affects MPN progression and biology. Therefore, the study of these cells can contribute not only to further clarifying their role in MPN and why they develop mechanisms of immune escape but also to favoring the development of new therapeutic strategies based on the restoration of antitumoral immune defenses.

In this context, our study aimed to evaluate the frequency, receptor expression, maturation profile, and function of NK cells from a conditional Jak2V617F murine transgenic model and MPN patients from a tertiary care hospital.

## 2. Methods

### 2.1 Study approval

Animal studies were approved by the Animal Use Ethical Committee (070/2015), and human peripheral blood (PB) samples were obtained after written informed consent and approval by the institutional review board of the University Hospital Ethical Committee (1.639.214), both at Ribeirao Preto Medical School, University of Sao Paulo, Brazil.

### 2.2 Samples

#### 2.2.1 Mice

Heterozygous wild-type/flox mice harboring the silent *Jak2*V617F mutation (Jak2WT/VF) previously described (34) were bred to vav-iCre mice (35), both in the C57BL/6 background, to generate the Jak2V617F-vav-iCre transgenic mice in which the *Jak2* mutation was specifically expressed in the hematopoietic system and endothelial cells. Exposure to Cre recombinase results in inversion of the mutant exon followed by an excision reaction that removes the WT exon, one LoxP site, and one LoxP511 site, fixing the inversion in place, thus generating Jak2WT/VF Cre^+^ mice (from here on, Jak2VF) that develop a lethal myeloproliferative neoplasm with 100% penetrance as the original model. The Jak2V617F and the Vav-i-Cre original mice were donated by Dr. Ann Mullaly and Dr. Daniel G. Tenen, respectively, both from Harvard Medical School. The colonies were expanded, and mice were kept in the Laboratory of Animal Experimental Studies, located at the Regional Blood Center building at the University of Sao Paulo, under specific pathogen-free conditions.

#### 2.2.2 Human

Eighty-four PB samples were obtained from patients with classical MPN diagnosis (29 with PV, 30 with ET, and 25 with PMF), of which 54 were JAK2V617F mutated and 15 were JAK2WT, aged 18 to 85 years old, from the Hematology Service at Ribeirao Preto Medical School of the University of Sao Paulo. Fifty-five PB samples from healthy individuals were obtained and processed as controls. Details on the number of patients included in each test are described in the corresponding figure legend.

### 2.3 Flow cytometry

**Figure 1:**
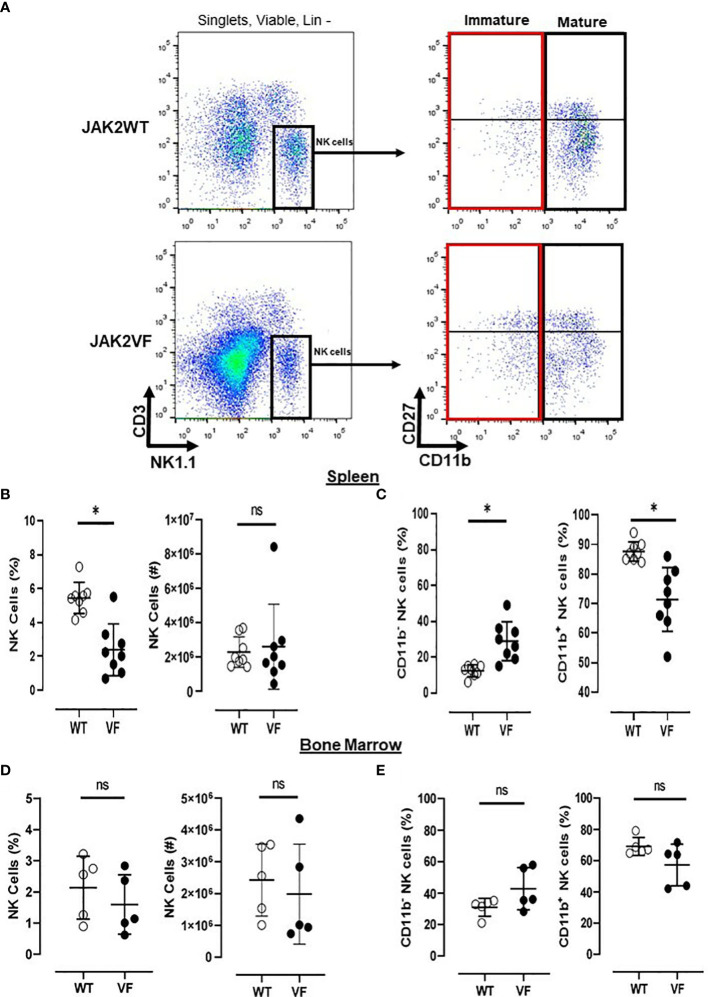
Mature NK cells are low numbered in Jak2V617F mice. **(A)** Gating strategy to characterize NK cells (Lin neg(^−^) [TER119^−^/CD4^−^/CD8^−^/CD19^−^] CD3^−^ NK1.1^+^) and their maturational subsets, in myeloproliferative neoplasms with JAK2V617F mutation (VF) or not (WT), defined as mature (CD27^−^CD11b^+^ and CD27^+^CD11b^+^) and immature (CD27−CD11b− and CD27^+^CD11b^−^) NK cells. Frequency and absolute numbers of NK cells in **(B)** spleen and **(D)** bone marrow. Frequency of immature (CD11b^−^) and mature (CD11b^+^) NK cells in **(C)** spleen and **(E)** bone marrow. VF, JAK2V617F mutated (n = 13); WT, JAK2 wild-type (n = 13). Error bars indicate the mean (± SEM). NS, not significant (p > 0.05); *p < 0.05; Mann–Whitney tests.

Single-cell suspensions from spleen and bone marrow murine cells were isolated, submitted to red cell lyse, and stained with fluorescence-conjugated antibodies (BioLegend, USA) against biotin TER-119/erythroid cells (clone: TER119), biotin CD19 (clone: MB19-1), biotin CD4 (clone: GK1.5), biotin CD8 (clone: 53-6.7), APC/CY7 CD3 (clone: 145-2C11), APC NK1.1 (clone: PK 136), FITC CD27 (clone: LG.3A10), and PE CD11b (clone: M1/70). To select NK cells, doublets, residual erythrocytes, and B and T lymphocytes (negative lineage: Lin^−^) were excluded, followed by gating on viable cells, Lin^−^ CD3^−^ NK1.1^+^, CD11b^−^ NK cells (CD27^−^CD11b^−^ and CD27^−^), and CD11b^+^ NK cells (CD27^+^CD11b^+^ and CD11b^+^) (**Figure 1A**).

Human PB cells were stained with anti-CD19-FITC (clone HIB19), anti-CD16-PE (clone 3G8), anti-CD56-PE (clone 159), anti-CD57-FITC (clone HNK-1), anti-CD45-PercP (clone 2D1), anti-CD3-APC (clone UCHT1), anti-CD11b-BV421 (clone ICRF44), and anti-CD27-BV510 (clone L127) antibodies (all from BD Biosciences, USA), as well as with isotype controls. An average of 100,000 events was acquired. The NK-cell phenotype was determined as CD19^−^/CD3^−^/CD56^+^ within the lymphocytes gate, and CD56^+^ NK cells were stratified into classical and maturational subsets, as determined by CD56/CD16 or CD27/CD11b (**Supplementary Figure S1A**) staining, respectively. The correspondence of simultaneous CD56^dim^CD16^+^ classical subset and CD11b^+^ mature and cytotoxic subpopulation with the equivalence of activating and inhibitory receptor expression is shown in **Supplementary Figure S1B**. To better determine maturation stage, three phenotypes were considered based on the expression of CD57: CD56bright CD16^−^CD57^−^ (immature), CD56^dim^CD16^+^CD57^−^ (intermediate), and CD56^dim^CD16^+^CD57^+^ (mature) NK cells (**Supplementary Figure S1C**). To quantify NK receptors, NKG2D-APC (clone 1D11, BD Biosciences, USA), NKp46-PECy7 (clone 9-E2, BD Biosciences, USA), NKG2A-APC (clone DX22, BioLegend, USA), and KIR2DL-PECy7 (clone DX27, BioLegend, USA) were used.

Data acquisition was performed on a FACS Canto II (BD Biosciences, USA), and FlowJo version X software was used for analysis.

### 2.4 Activation, degranulation, and cytotoxicity assays

For activation and degranulation assays in murine samples, 8-week-old female mice (WT, *n* = 4; VF, *n* = 4) were used, and sorted NK cells were stimulated with a YAC-1 cell line (10:1 E:T ratio) for 3 h and evaluated for CD69 and CD107a expression, respectively.

For the functional NK assays from MPN patients, peripheral blood mononuclear cells (PBMC) were isolated by Ficoll–Hypaque gradient centrifugation (*d* = 1.077, Sigma-Aldrich, Darmstadt, Germany), counted, and assessed for viability using a trypan blue dye (0.2% in PBS). After quantification of NK by flow cytometry (CD45^+^/CD19^−^/CD3^−^/CD56^+^), the frequencies of NK cells among PBMC were calculated to prepare cellular suspensions containing NK cells, which were co-cultured with target cells for 2.5 h, in the presence of 100 U/ml of human IL-2 (PeproTech, USA) at 37° and 5% CO_2_. K562 cells (human myelogenous leukemia cell line), used as target cells, were cultured in RPMI 1640 medium supplemented with 2 mM of l-glutamine and 100 U/ml of penicillin, at 37°C in 5% CO_2_. Specific target cell death was determined by gating on SSC-A/CTO PE-A, settled to assess K562 cells, followed by an FSC-A/7-AAD PerCP plot, within the CTO+ target cell population (**Supplementary Figure S2**). To assess NK degranulation, a ratio of 50:1 of effector (E) and target cells (T) was used, and antihuman CD107a and 7AAD staining were performed. To evaluate NK cytotoxicity, an E:T ratio of 25:1 was chosen and 5 µl of CellTracker Orange CMTMR (CTO, Invitrogen, Eugene, OR, USA) was used to label target cells for 1 h at 37°C and 5% CO_2_. K562 cells were gated as CTO^+^ events, and dead K562 were then gated to calculate the percentage of specific death by calculating: % specific death − % spontaneous death/100 − % spontaneous death. Data were acquired in a FACS Canto II and analyzed using the FlowJo software.

### 2.5 Analysis of *Jak2* gene expression in murine NK cells

Total RNA was extracted from sorted NK cells using Trizol. The quantification and purity of RNA were evaluated at 260 and 280 nm using a DS-11 spectrometer (Denovix, USA). cDNA was synthesized using High Capacity (Applied Biosystems, Foster City, CA, USA) and real-time RT-PCR was performed using a 7500 Real-Time PCR System (Applied Biosystems, Foster City, CA, USA) with Power Sybr Green Master Mix (Applied Biosystems, Foster City, CA, USA). The constitutive gene mGAPDH was used as the normalizer of expression to calculate the cDNA copy numbers of the genes. PCR conditions were as follows: 95°C for 15 s, followed by 40 cycles of 95°C for 10 s and 60°C for 30 s. Each sample was measured in duplicate, and the comparative threshold cycle method was used for the relative quantification of gene expression. Primer sequences were as follows: Jak2WT (Forward primer): 5′TTTGAATTATGGTGTCTGTG 3′; Jak2V617F (Forward primer): 5′TTTGAATTATGGTGTCTGTT 3′; ak2 (Reverse primer): 5′CAGGTATGTATCCAGTGATCC 3′.

### 2.6 Cell proliferation

After culturing in six-well plates in RPMI1640 medium conditioned with 10% of FBS and 1,000 U/ml of recombinant murine IL-2 (PeproTech, USA) for 72 h, 2 million NK cells isolated from Jak2WT or Jak2VF animals were collected and fixed with 70% ethanol and stored at −20°C for 24 h. Ki-67 staining was performed following the manufacturer’s instructions (Ki-67 PE clone SolA15; BioLegend, USA). Next, the mean fluorescence intensity (MFI) was obtained by flow cytometry using standard techniques in a FACSCalibur instrument (BD Biosciences, USA). IgG isotype was used as a negative control for each condition.

### 2.7 Sorting of murine NK cells and HSC

Immunomagnetic depletion of T lymphocytes, B cells, and residual erythrocytes using biotinylated antibodies anti-TER119, anti-CD19, anti-CD4, and anti-CD8, by negative selection, was used out to enrich the sample for NK cells, which were sorted as lineage negative (anti-streptavidin, STV^−^), CD3^−^, and anti-NK1.1^+^. For HSC isolation, immunomagnetic depletion cocktail included biotinylated anti-TER119, anti-CD19, anti-CD8, anti-CD11b, anti-B220, anti-Gr1e anti-CD3 antibodies, and then STV^−^ (or Lin^−^) Sca-1^+^cKit^hi^SLAM^+^ (LSK SLAM^+^) cells were sorted. The viability marker DAPI was added to stained cells, which were subsequently taken to the Cell Sorter FACSAriaII™ or Cell Sorter FACSAria Fusion™ (BD Biosciences, USA) to isolate the cells of interest.

### 2.8 Co-culture conditions

After sorting CD45.2^+^ murine NK and CD45.1^+^ HSC cells (LSK SLAM^+^), co-culture of NK : HSC cells in a 100:1 ratio was carried out. Cells were plated in StemSpan SFEM Medium^®^ supplemented with IL-2 (1,000 U/ml), SCF (100 ng/ml), IL-3 (10 ng/ml), IL-6 (10 ng/ml), FLT3 (100 ng/ml), and TPO (25 ng/ml), as well as penicillin and streptomycin (1%) for 24 h. This ratio was proposed to simulate the average proportion of NK and HSC observed in the healthy bone marrow. After that, cells were seeded in methylcellulose (MethoCult M3534, StemCell Technology, Canada) at a concentration of 500 HSC per well in biological duplicates. The methylcellulose plates were incubated at 37°C for 12–15 days. For analysis, the averages of the number of colonies observed in the duplicates of each experimental condition were considered (**Figure 8A**).

## 3. Results

### 3.1 Murine Jak2V617F NK cells present an immature immunophenotypic profile when compared to normal NK cells

When compared to the control group, Jak2VF mice presented a lower relative frequency of NK cells in the spleen, although absolute numbers were not significantly different (**Figure 1B**). Bone marrow NK frequency was similar in both groups (**Figure 1D**).

**Figure 2:**
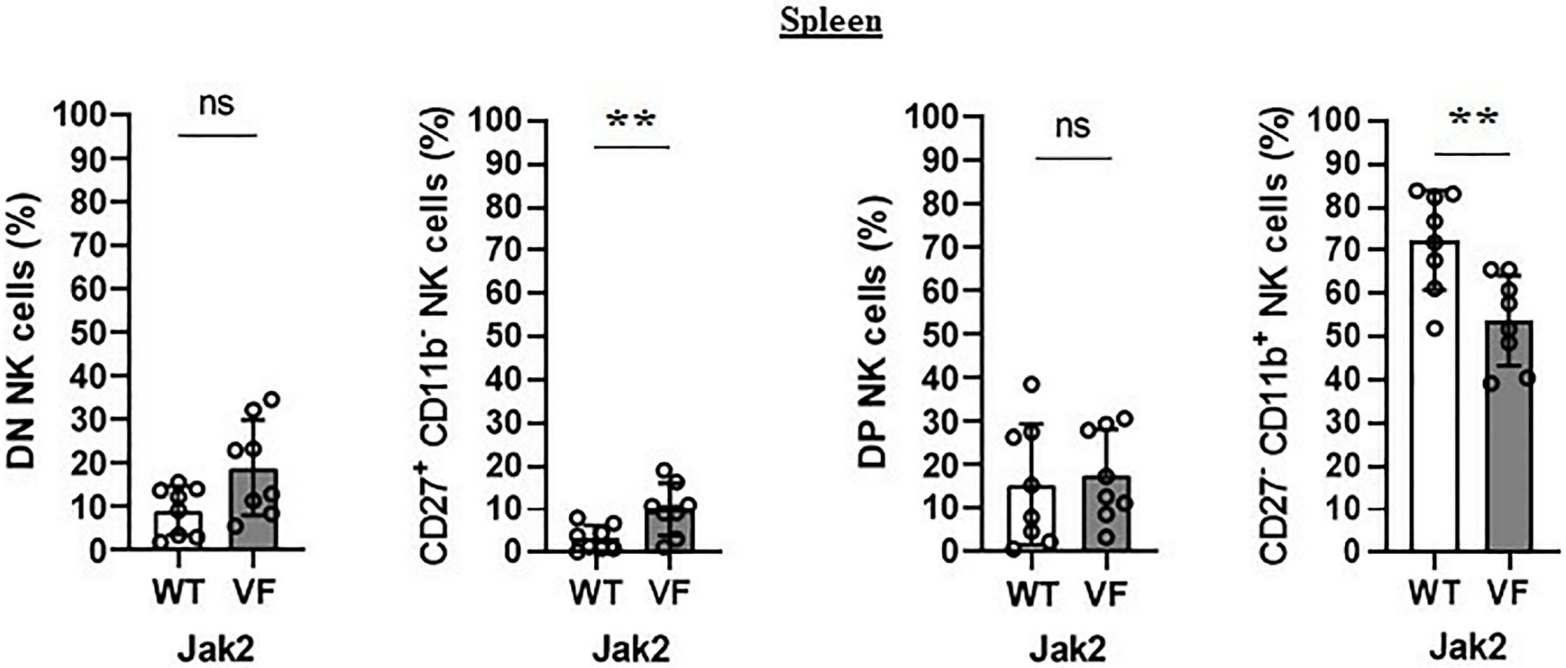
The maturation arrest of Jak2V617F murine NK cells is associated with an imbalance between CD27^+^ and CD11b^+^ NK subsets. The frequency and absolute number of CD27^−^CD11b^−^ [double negative (DN)], CD27^+^CD11b^−^, CD27^+^CD11b^+^ [double positive (DP)], and CD27^−^CD11b^+^ NK cells. VF, Jak2V617F mutated (n = 8); WT, Jak2 wild-type (n = 8). Error bars indicate the mean (± SEM). NS, not significant (p > 0.05); **p < 0.01; Mann-Whitney test.

The maturation profile analysis showed an increased frequency of immature CD11b^−^ NK cells and a decrease of mature CD11b^+^ NK cells in the spleen of Jak2VF mice as compared to controls (**Figure 1C**). The imbalance between CD11b^-^ NK cells and CD11b^+^ NK cells in Jak2V617F animals was associated with an almost threefold increase in the relative frequency of CD27^+^ more immature NK cells as well as a twofold increase of the CD27^−^CD11b^−^ subset (double negative (DN)). In agreement, a deep reduction in the frequency of the cytotoxic subset CD11b^+^CD27^−^ NK cells was observed (**Figure 2**). A similar trend of distribution of Jak2VF NK subsets was found in the bone marrow as compared to the findings in the spleen (**Figure 1E**).

### 3.2 NK cells are frequent although immature in MPN patients

As expected, MPN patients presented lower relative and absolute frequencies of total lymphocytes than controls, particularly in PMF. This lower lymphocyte frequency in MPN patients as a group (including PV, ET, and PMF) was noted regardless of the JAK2V617F mutation (**Supplementary Figure S3**).

**Figure 3:**
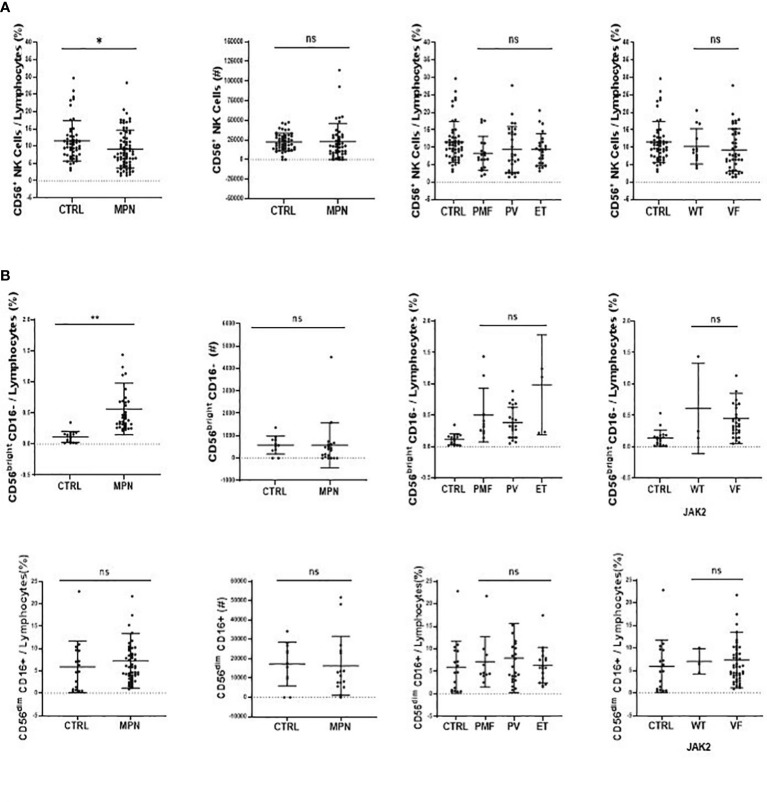
CD56bright CD16^−^ NK-cell subset is increased inMPN patients. The frequency and absolute number of **(A)** NK cells (CD45high, CD19^−^, CD3^−^, CD56^+^) and **(B)** classical NK function subsets (CD56brightCD16^−^ and CD56dimCD16^+^). CTRL, control (n = 17); MPN, myeloproliferative neoplasms (n = 49); PV, polycythemia vera (n = 22); ET, essential thrombocythemia (n = 5); PMF, primary myelofibrosis (n = 10). Error bars indicate the mean (± SEM). NS, not significant (p > 0.05); *p < 0.05; **p < 0.01; Mann–Whitney and Kruskal–Wallis tests.

When compared to controls, there was a higher frequency and number of CD56^+^ NK cells in MPN blood samples among total lymphocytes. This was true for all disease subtypes. It is worth noting that MPN JAK2VF samples presented more CD56^+^ NK cells than JAK2WT and control samples (**Figure 3A**).

**Figure 4:**
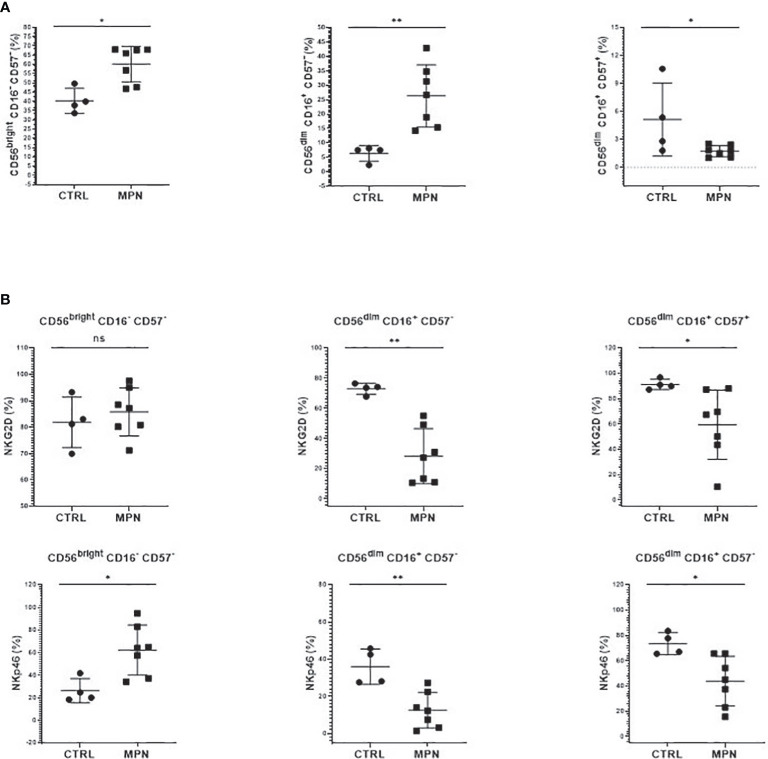
NK cells from MPN patients are phenotypically immature. The frequency of **(A)** immature (CD56^bright^CD16^−^CD57^−^), intermediate (CD56^dim^CD16^+^CD57^−^), and mature (CD56^dim^ CD16^+^ CD57^+^) NK cells; **(B)** NK-cell activation (NKG2D, NKp46) receptors by CD57 maturation subsets. CTRL, control (n = 4); MPN, myeloproliferative neoplasms (n = 7). Error bars indicate the mean (± SEM). NS, not significant (p > 0.05); *p < 0.05; **p < 0.01; Mann–Whitney and Kruskal–Wallis tests.

**Figure 5:**
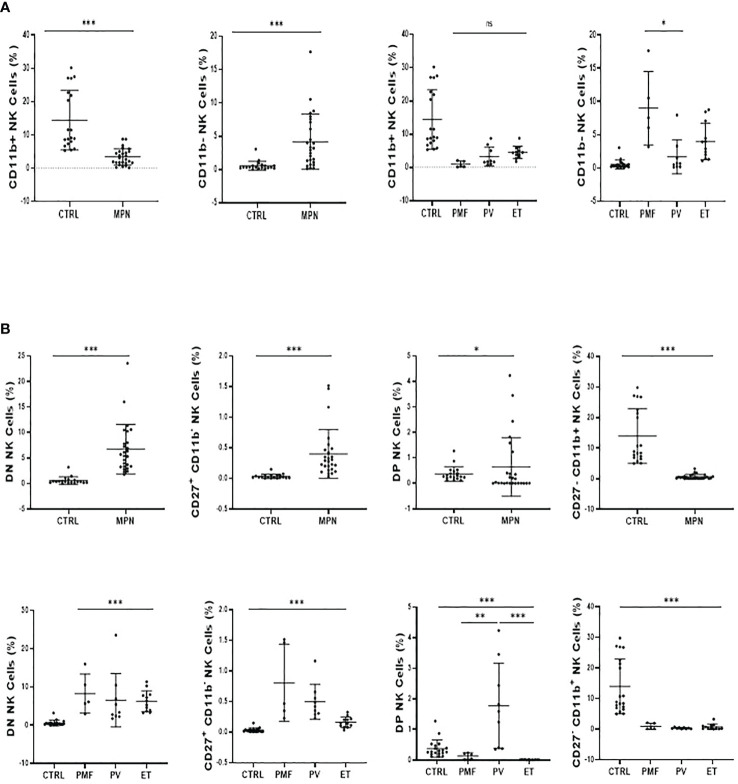
MPN patients show an imbalance between CD11b^+^ and CD27+ NK-cell subsets. **(A)** The frequency of CD11b^+^ (Mature) and CD11b^−^ (Immature) NK cells subsets and; **(B)** the frequency and absolute number of CD27^−^CD11b^−^ [double negative (DN)], CD27^+^CD11b^−^, CD27^+^CD11b^+^ [double positive (DP)], and CD27^−^CD11b^+^. NK-cell frequency and maturation profile in myeloproliferative neoplasms according to disease subtype and JAK2V617F mutation (WT or VF). CTRL, control; MPN, myeloproliferative neoplasms (n=26); PV, polycythemia vera (n = 9); ET, essential thrombocythemia (n = 12); PMF, primary myelofibrosis (n = 5). Error bars indicate the mean (± SEM). NS, not significant (p > 0.05); *p < 0.05; **p < 0.01; ***p < 0.001; Mann–Whitney and Kruskal–Wallis tests.

Although the frequency of NK cells among lymphocytes in MPN was increased when compared to healthy subjects, a maturation profile defect was observed. An increased frequency of immature CD56^bright^ CD16^−^ secretory NK cells was observed in MPN patients, but no difference was found regarding the CD56^dim^CD16^+^ subset (**Figure 3B**). When looking into the expression of CD57 among the CD56^dim^ and CD56^bright^ populations, we also found an increased frequency of an immature NK-cell subset that slightly decreased when matured to the intermediate subset but remained high compared to normal and significantly decreased in the most mature and allegedly cytotoxic subset (**Figure 4A**). In line with previous results, a decreased expression of activating receptors NKG2D and NKp46 was found in both intermediate and mature CD57 subsets (**Figure 4B**). The gating strategy that shows the phenotypical equivalence of CD11b^+^ and CD56^dim^ NK cells and the maturation stages by expression of CD57 is detailed in **Supplementary Figure S1C**. In agreement with that, we found an increased immature CD11b^−^ NK-cell population and a decrease in more mature CD11b^+^ NK cells when compared to the control group. Such imbalance towards a more immature phenotype was more evident in PMF among the MPN subtypes, especially when compared to PV (**Figure 5A**). When all the functional subtypes were characterized, MPN samples presented a higher frequency of DN (CD27^−^CD11b^−^) NK cells and an increased frequency of CD27^+^ NK cells. Among MPN subtypes, PMF samples had the highest frequency of both DN NK and CD27^+^ NK cells. Finally, the frequency of double-positive (DP, CD27^+^CD11b^+^) NK cells was higher in PV when compared to PMF and ET (**Figure 5**).

**Figure 6:**
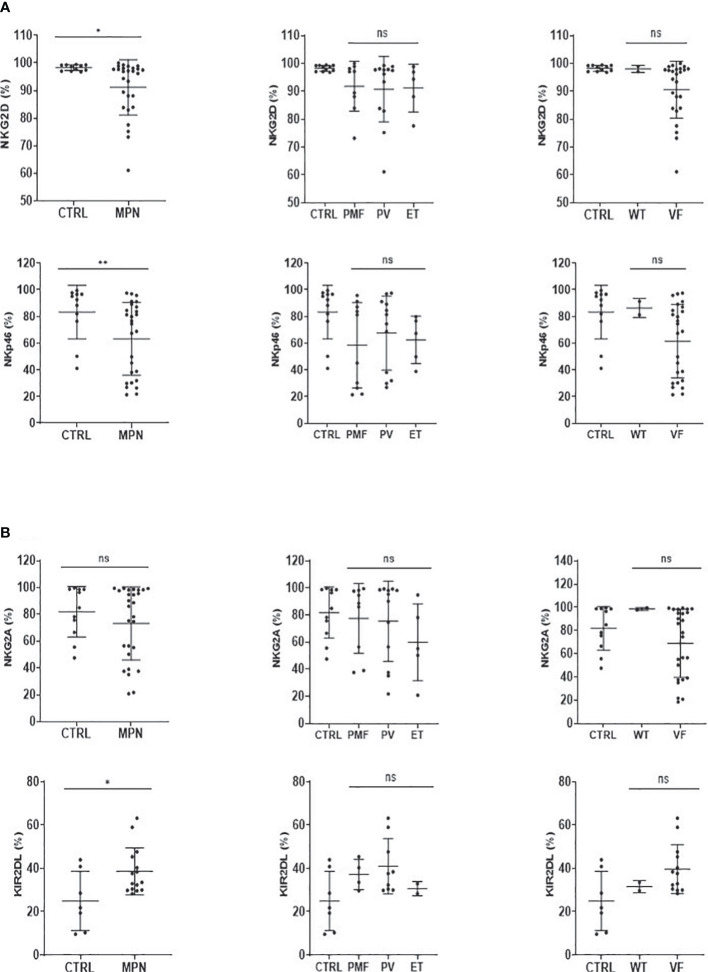
NK-cell activatory and inhibitory receptors are imbalanced in MPN patients. The frequency of **(A)** NK-cell activation (NKG2D, NKp46) and **(B)** NK-cell inhibition (NKG2A, KIR2DL) receptor cells in myeloproliferative neoplasms with JAK2V617F mutation (VF) or not (WT). CTRL, control; MPN, myeloproliferative neoplasms (n = 27); PV, polycythemia vera (n = 12); ET, essential thrombocythemia (n = 5); PMF, primary Myelofibrosis (n = 10). Error bars indicate the mean (± SEM). NS, not significant (p > 0.05); *p < 0.05; **p < 0.01; Mann–Whitney and Kruskal–Wallis tests.

The expression of NK receptors was also investigated. We observed that the frequency of both NKG2D and NKp46 activation receptors was decreased in MPN (**Figure 6A**), while the KIR2DL inhibitory receptor was increased when compared to controls (**Figure 6B**). Although visually MPN patients harboring the JAK2V617F mutation showed lower expression of NKG2D and NKp46, no significance was found (**Figure 6A**).

### 3.3 NK dysfunction in the Jak2-mutated murine model is dependent on the V617F mutation

mRNA from murine NK cells sorted from Jak2WT (*N* = 4) and Jak2VF (*N* = 3) mice were obtained and analyzed by RT-PCR to investigate if the Jak2V617F mutation was detected in NK cells. We observed that NK cells from Jak2VF mice express the V617F mutation as well as lower levels of the wild-type gene when compared to controls (**Supplementary Figures S4A, B**).

### 3.4 Jak2V617F NK cells are dysfunctional

**Figure 7:**
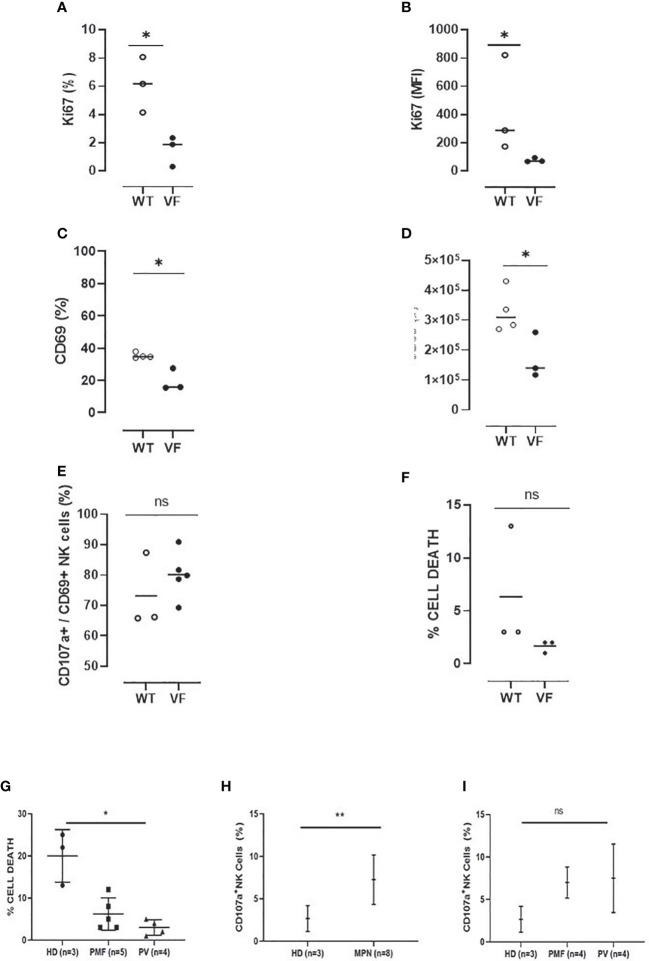
Murine and human NK cells from MPN present defective activation and cytotoxicity as compared to control NK cells. **(A)** The frequency and **(B)** mean fluorescence intensity (MFI) of Ki-67 in Jak2WT (n = 4) and Jak2VF (n = 3) NK cells after 72 h of culture in the presence of IL-2. **(C)** The proportion and **(D)** number of activated Jak2WT and Jak2VF NK cells (CD69^+^). **(E)** The frequency of CD69+ NK cells expressing the degranulation marker CD107a after 3 h of stimulation with YAC-1. **(F)** The frequency of cell death after YAC-1 stimulation. **(G)** The frequency of cell death and **(H–I)** NK degranulation in myeloproliferative neoplasms with JAK2V617F mutation (VF) or not (WT), measured by CD107a expression after stimulation with the K562 target cell. VF, Jak2V617F mutated; WT, Jak2 wild-type; CTRL, control (n = 3); MPN, myeloproliferative neoplasms (n = 9); PV, polycythemia vera (n = 4); PMF, primary myelofibrosis (n = 5). Error bars indicate the mean (± SEM). NS, not significant (p > 0.05); *p < 0.05; **p < 0.01; Mann–Whitney and Kruskal–Wallis tests.

After characterization of NK cells, experiments were carried out to evaluate their effector functions. First, the proliferative capacity of splenic NK cells was evaluated after stimulation with IL-2 for 72 h. These experiments indicated that Jak2VF NK cells were less responsive to the cytokine, resulting in a lower frequency of proliferating cells and reduced expression of Ki-67 (**Figures 7A, B**).

The effector properties of NK cells were evaluated by characterizing the expression of CD69 and CD107a after stimulation with PMA and ionomycin. When compared to the control group, there was a reduction in the frequency and absolute numbers of CD69 expressing NK cells in Jak2VF animals (**Figures 7C, D**). In addition, even though not significant, a trend of decreasing degranulating CD69^+^CD107a^+^ NK cells and target cell killing was observed in the mutated group when compared to the control group (**Figures 7E, F**).

Human NK-cell cytotoxicity ability and expression of CD107a after interaction with an NK-cell–sensitive target (K562 cell line) were evaluated. As compared to controls (health donor (HD)), MPN patients had less cytotoxic activity, and PV patients showed a significant reduction in NK killing property. On the contrary, the degranulation assay showed a higher frequency of CD107a membrane expression in MPN patients when compared to controls, indicating that NK cells are able to degranulate but do not kill target cells effectively. No difference in degranulation was found between PV and PMF patients (**Figures 7G–I**).

### 3.5 Jak2V617F NK cells can regulate HSC clonogenic potential and differentiation

**Figure 8:**
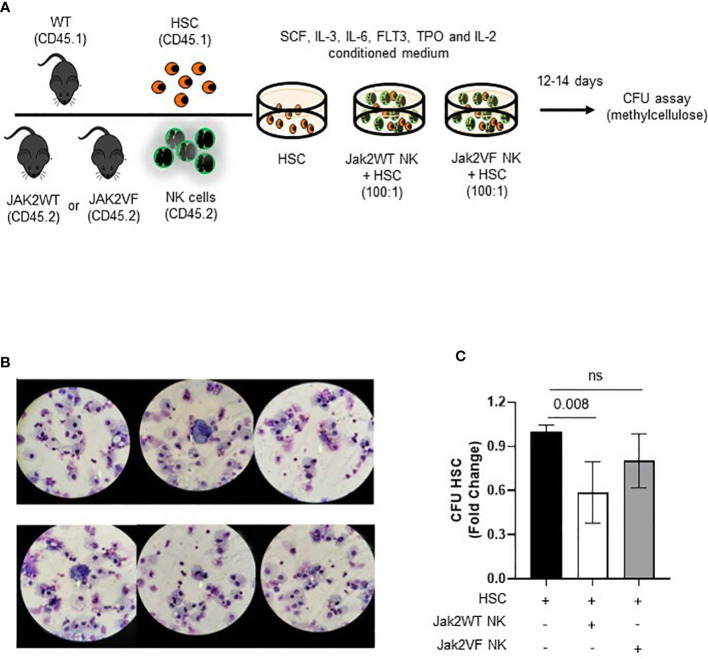
NK cells from Jak2V617F mice are deficient in regulating the differentiation and clonogenic capacity of hematopoietic stem cells (HSC). **(A)** Schematic figure of the strategy used to study the NK-HSC interaction in culture. **(B)** Illustrative image of co-culture cytospins showing HSC (LSK SLAM^+^ cells) differentiation after 12 days of methylcellulose colony assay. The majority of CFU-derived cells corresponded to macrophages and granulocytic progenitors. **(C)** Graphic representation of the quantification of colony forming units (CFU) expressed as fold change in the following conditions: HSC, Jak2WT NK cells + HSC, Jak2VF NK cells + HSC. VF: Jak2V617F mutated; WT: Jak2 wild-type. CTRL: Control; MPN: myeloproliferative neoplasms. PV: polycythemia vera; ET: essential thrombocythemia; PMF: primary myelofibrosis. Error bars indicate the mean (± SEM). NS, not significant (*p* < 0.05), Kruskal Wallis test.

The findings of phenotypic and functional changes in NK cells from the MPN model used in this study prompted us to assess the impact of these changes on LSC regulation. For this, LSK SLAM^+^CD45.1^+^ cells (LT-HSC), and Jak2WT or Jak2VFCD45.2^+^ NK cells were isolated by sorting, cultured for 24 h, and then taken to colony-forming units (CFU) assays (**Figure 8A**). In total, 500 LT-HSC were exposed or not to 50,000 Jak2WT or Jak2VF NK cells for 24 h and then plated in methylcellulose. After a 12-day co-incubation, colony counts showed that LT-HSC was able to differentiate in all the experimental conditions (**Figure 8B**). When compared to nonexposed, NK-exposed HSC gave rise to a reduced number of colonies. HSC + Jak2WT NK co-cultures presented reduced colony formation by 42%, as compared to HSC-only cultures, while HSC + Jak2VF NK co-cultures decreased by 20% the number of HSC colonies (**Figure 8C**), indicating that Jak2-mutated NK cells were not able to reduce the HSC clonogenic potential as normal NK cells in cell culture assay models.

## 4. Discussion

Although useful to target the disease phenotype, the failure of JAK2 pharmacological inhibitors to eliminate LSC in BCR-ABL1-negative MPN suggests that targeting other elements associated with MPN pathogenesis is crucial. Extrinsic factors, such as tumor microenvironment and the influence of other cells on HSC and hematopoietic precursors, may also have a role in the pathogenesis of the disease. The immune system dysregulation is key to the pathology of MPN, supporting clonal evolution, mediating symptoms, and resulting in varying degrees of immunocompromise. Cytokine levels and the immune phenotype in patients differ from those in healthy donors due to the driver mutations identified in MPN, as they all affect the JAK-STAT signaling pathway. In that scenario, the growing knowledge regarding the role of NK-cell phenotype and function and also the mechanisms through which they act in the pathophysiology and contribute to immune escape from cancer and MPN surveillance may contribute to the development of new therapeutic strategies (36).

In the present study, we characterized NK cells in MPN samples from patients and mice from a conditional knock-in model of the heterozygous expression of the Jak2V617F mutation to investigate the role of NK-cell activity in the pathogenesis of the disease. The immunophenotypic characterization of spleen and bone marrow NK cells of Jak2VF animals revealed differences in maturation and function when compared to wild-type controls. Mice carrying the Jak2V617F mutation presented splenomegaly and a lower frequency of NK cells, resulting in the reduced representativeness of the lymphoid compartment and NK cells as expected by the myeloid expansion that characterizes MPN. In contrast, human samples presented a higher frequency of NK cells than JAK2V617F-mutated MPN samples, but no differences were found in the absolute numbers in humans or mice.

Despite the absence of NK absolute reduction, we demonstrated compromised maturation of NK cells in Jak2VF-mutated animals that exhibit an increase of immature NK cells and a decrease of mature cytotoxic NK cells. Indeed, even though NK cells were increased in MPN patients according to the classical CD56/CD16 expression, their maturation profile was also unbalanced, with higher noncytotoxic CD11b^−^ cells and lower mature CD11b^+^ cells. CD11b and CD27 have been more recently recognized as biomarkers of NK-cell maturation, also in human NK cells, which prompted us to include them for human NK characterization in MPN patients. Among MPN entities, both CD11b^+^ and CD11b^−^ NK cells were decreased as compared to healthy controls in PV and ET while PMF patients presented not only a reduced cytotoxic phenotype but also an imbalance towards an immature NK profile with a significant increase in CD11b^−^ cells as well as CD11b^−^CD27^+^ NK cells. Therefore, PMF samples from patients and Jak2-mutated primary NK cells were shown to have a more secretory NK-cell pattern, which may contribute to the important inflammatory profile of the disease. Functionally, regardless of increased degranulation (higher frequency of CD107a), NK cells from MPN patients had lower cytotoxicity ability. CD107a expression correlates with both cytokine secretion and NK-cell–mediated lysis of target cells. Therefore, MPN patients display an immature phenotype with high secretory ability that might be stimulating degranulation but not functioning for killing the target cell.

In addition, the results indicated that JAK2V617F NK cells are less sensitive to activation (by the lower expression of CD107a and CD69 functional markers) and proliferation after stimulation, thus reinforcing that, in addition to the maturation arrest, NK cells are also dysfunctional in MPN.

Mature NK cells are mainly located in the spleen and peripheral blood. Their role in tumor surveillance is facilitated by the recirculation of mature and more functional NK cells from the spleen to the blood and then back to the spleen, which increases the efficiency of the immune system to recognize and eliminate transformed cells (37, 38).

Considering that the frequency and functional maturation of NK cells are relevant for normal HSC properties, the maturation arrest here found may favor MPN progression due to uncontrolled expansion of the neoplastic HSC (39). In agreement, the co-culture of normal HSC and Jak2-mutated dysfunctional NK cells resulted in less impairment of HSC clonogenicity when compared to the colony number reduction observed in similar cultures using Jak2WT NK cells. We believe that the phenotypic and functional changes of MPN NK cells have an impact on HSC regulation.

Our findings are corroborated by some studies showing that phenotypic and functional impairment of NK cells is associated with hematological neoplasms and their prognosis, including response to treatment, overall survival, and a greater predisposition to viral infections (23, 40, 41). In myelodysplastic syndrome patients, for example, it has been shown that NK-cell defects are, in most cases, attributed to a defective differentiation of these cells that may compromise tumor immune surveillance and accelerate disease progression (42). The impact of the NK-cell differentiation profile has been demonstrated in a model of leukemic blast transplantation in which tumor progression and graft-versus-leukemia effects were attributed to the maturation of NK cells (43). Another work showed deficient NK cell development through hyperactivation of the aryl hydrocarbon receptor after contact with isolated myeloid leukemia blasts, turning them less sensitive to maturation and activation by IL-15, which led to an accumulation of immature cells with little functional capacity (44).

As expected by the use of the VAV-iCRE model that drives the Jak2 expression to the hematopoietic compartment, we expected that the V617F mutation was present in hematopoietic cells. However, it was not clear in the literature if NK cells themselves expressed the mutation since the JAK2 mutation drives a myeloid and not lymphoid expansion in MPN. Other authors have described the expression of Jak2V617F in lympho-myeloid stem/progenitor cells (45, 46). Iida and colleagues (47) described reduced numbers of lymphoid progenitors with functionally competent B lineage preserved in a Jak2V617 model but, again, mature NK cells were not explored. Here, we demonstrated the expression of Jak2V617F in murine NK isolated cells from a conditional murine model. This result suggests that the phenotypic dysfunction observed in NK cells is dependent on the Jak2 mutation, which supports our findings in MPN patients harboring the mutation.

In conclusion, exacerbated hematopoiesis is a hallmark of MPN and affects one or multiple myeloid cells. Still, it is not clear to what extent lymphopoiesis is also affected by the impaired differentiation capacity of aberrant hematopoietic progenitor cells or a compromised stem cell microenvironment. Both mechanisms can potentially explain our observations that NK cells show an immature profile with deficient cytotoxicity, leading to impaired tumor surveillance in JAK2V617F-mutated MPN. Our observations provide further insight into the complexity of the mechanisms of action of NK cells in myeloid malignancies. Exploring NK cells’ genetic profiles and signaling pathways can further clarify their role in myeloid malignancies as well as favor the development of new therapeutic strategies to control neoplastic stem cell function.

## Data availability statement

The original contributions presented in the study are included in the article/**Supplementary Material**. Further inquiries can be directed to the corresponding author.

## Ethics statement

The studies involving human participants were reviewed and approved by Research Ethics Committee of Ribeirao Preto Medical School of the University of Sao Paulo. The patients/participants provided their written informed consent to participate in this study. The animal study was reviewed and approved by Ethics Committee on the Use of Animals (CEUA).

## Author contributions

AFOC, LOM, TMB, AQA, RSW, and LLF-P conceived and designed the experiments. TMB, AQA, LOM, DAP-M, IAL, LSB, CAS, PSS, and JLSS performed experimental work. AFOC, LOM, TMB, PSS, SSK, LCP, JAM-N, and EMR contributed to the investigation or data analysis. AFOC, TMB, and LOM performed the statistical analysis and prepared the figures. AFOC, LOM, AQA, TMB, and LLF-P wrote the paper. All authors contributed to the article and approved the submitted version.

## Funding

This work was supported by grants from São Paulo Research Foudation – FAPESP (Grants #2015/21866-1 and #2021/06841-3) and National Council for Scientific and Technological Development – CNPq (INCT #465539/2014-9) to LLF-P. AFOC, LOM, TMB, IAL and LSB are/were supported by CAPES-Brazil fellowships.

## Acknowledgments

The authors would like to thank Prof. Daniel G. Tenen for providing the Vav-Cre transgenic mice, SK and Prof. Ann Mullally for the donation of Jak2V617F original mice, and Patricia Vianna Bonini Palma for the technical support at the sorting facility from the Regional Blood Center of Ribeirão Preto.

## Conflict of interest

The authors declare that the research was conducted in the absence of any commercial or financial relationships that could be construed as a potential conflict of interest.

## Publisher’s note

All claims expressed in this article are solely those of the authors and do not necessarily represent those of their affiliated organizations, or those of the publisher, the editors and the reviewers. Any product that may be evaluated in this article, or claim that may be made by its manufacturer, is not guaranteed or endorsed by the publisher.
