# Supplementary material for: Altered distribution and function of NK-cell subsets lead to impaired tumor surveillance in JAK2V617F myeloproliferative neoplasms
Source: Front Immunol. 2022 Sep 23;13:768592. doi: 10.3389/fimmu.2022.768592 (PMC9539129; doi:10.3389/fimmu.2022.768592)
Supplement: Supplementary file 1 [file DataSheet_1.pdf]

Supplementary Material

1 Supplementary Figures

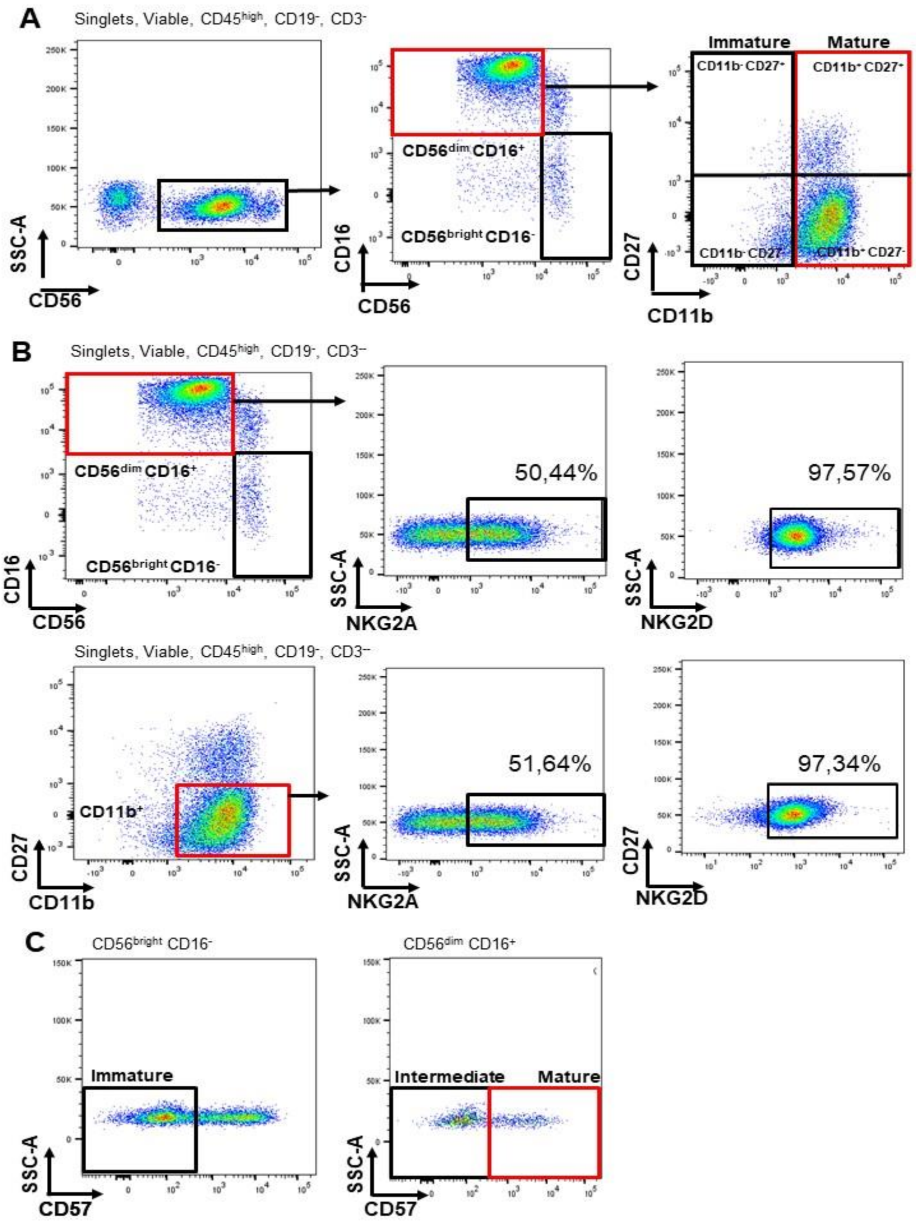

Supplementary Figure 1. Human CD11b$^+$ mature NK cell population corresponds to CD56^{dim} CD16$^+$ NK cells. Gating strategy of NK cells characterization (CD45^{high}, CD19$^-$, CD3$^-$, CD56$^+$) and (A) their classical functional subsets defined as cytotoxic (CD56^{dim} CD16$^+$) and secretory (CD56^{bright} CD16$^-$) and maturational subsets defined as mature (CD27$^-$CD11b$^+$ and CD27$^+$CD11b$^+$) and immature (CD27$^-$CD11b$^-$ and CD27$^+$CD11b$^-$); (B) the phenotypical equivalence of activating and inhibitory receptors expression by CD11b$^+$ mature NK subset and CD56^{dim} CD16$^+$ cytotoxic NK cells; and (C) the maturation profile of NK cell according to the expression of CD57 in immature (CD56^{bright} CD16$^-$ CD57$^-$), intermediate (CD56^{dim} CD16$^+$ CD57$^-$) and mature (CD56^{dim} CD16$^+$ CD57$^+$) NK cells.

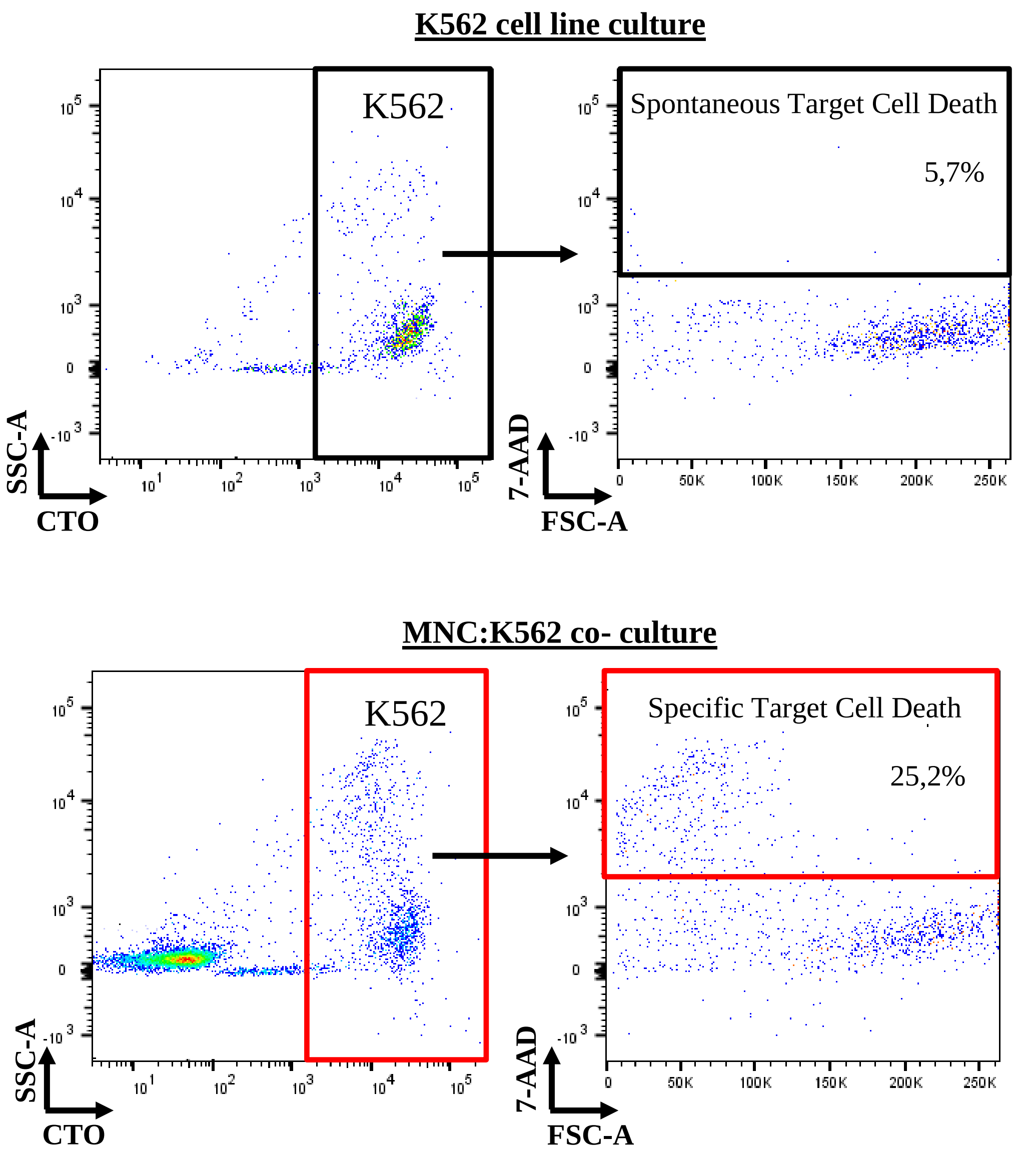

Supplementary Figure 2. Gating strategy for Specific Cell death frequency. First gate on SSC-A/CTO PE-A, settled to assess K562 cells, followed by a FSC-A/7-AAD PerCP gate, within the CTO+ target cell .population. MNC: Mononuclear cells.

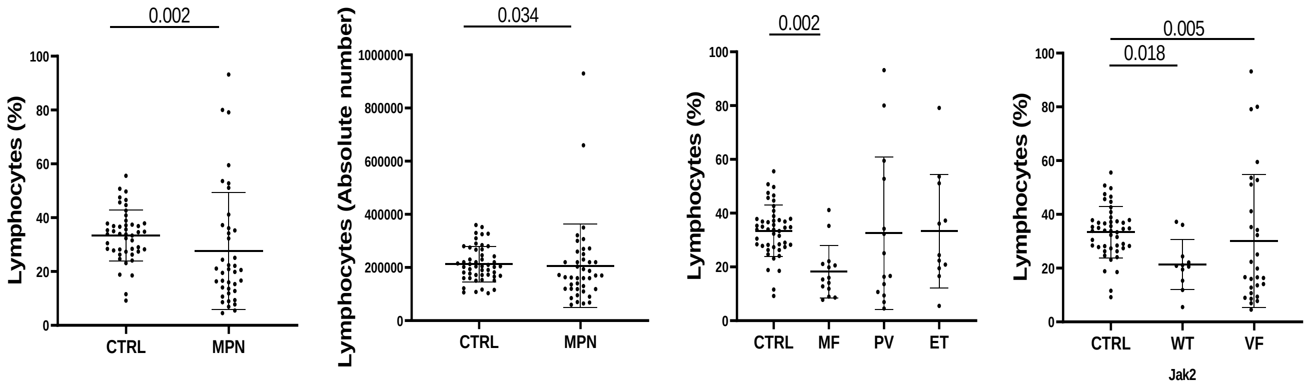

Supplementary Figure 3. Lymphocytes are low numbered in Myeloproliferative Neoplasms. Frequency and absolute numbers of lymphocytes in myeloproliferative neoplasms according to disease subtype and JAK2V617F mutation (WT or VF). CTRL: Control; MPN: Myeloproliferative neoplasms; PV: Polycythemia Vera; ET: Essential Thrombocythemia; PMF: Primary Myelofibrosis. Significance was demonstrated by p values or ns: not significant, Mann-Whitney and Kruskal Wallis tests.

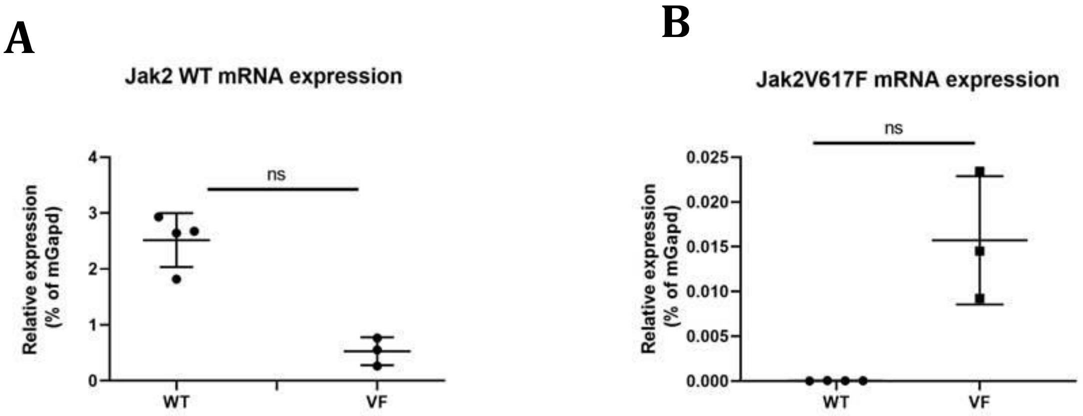

Supplementary Figure 4. Relative expression of Jak2 in Jak2WT and Jak2VF in murine sorted NK cells. Jak2 gene expression analysis was performed by RealTime PCR in Murine NK cells sorted from Jak2VF (n=4) and Jak2WT (n=3). mGapd was used to normalize and as a housekeeping gene. (A) Jak2WT expression is present in both NK cells, while (B) Jak2V617F expression is observed only in Jak2VF NK cells. Jak2VF: JAK2V617F mutated; JAK2 WT: wild-type. NS: not significant, Mann-Whitney test.
